# Reduced Visuospatial Attention in Personal Space is Not Limited to the Affected Limb in Complex Regional Pain Syndrome

**DOI:** 10.2147/JPR.S437366

**Published:** 2024-04-23

**Authors:** Monika Halicka, Olivia Rose Cousins, Antonia F Ten Brink, Axel D Vittersø, Michael J Proulx, Janet H Bultitude

**Affiliations:** 1 Institute of Neuroscience, Universite catholique de Louvain, Brussels, Belgium; 2 Centre for Pain Research, University of Bath, Bath, UK; 3 Department of Psychology, University of Bath, Bath, UK; 4 Department of Experimental Psychology, Helmholtz Institute, Utrecht University, Utrecht, the Netherlands; 5 Division of Mental Health Services, Akershus University Hospital, Lørenskog, Norway

**Keywords:** complex regional pain syndrome, spatial attention, personal space, pseudoneglect, temporal order judgement, chronic pain

## Abstract

**Purpose:**

Alterations in spatial attention have been reported in people with chronic pain and may be relevant to understanding its cortical mechanisms and developing novel treatments. There is conflicting evidence as to whether people with Complex Regional Pain Syndrome (CRPS) have reduced visuospatial attention to their affected limb and/or its surrounding space, with some evidence that these deficits may be greater in personal (bodily) space. We aimed to test the competing hypotheses of whether the visuospatial attentional bias is specific to the personal space of the affected limb or generalizes to the personal space of other parts of the affected side of the body.

**Patients and Methods:**

Using visual Temporal Order Judgement tasks, we measured spatial attention in the personal space of the hands and feet of patients with upper (n=14) or lower (n=14) limb CRPS and pain-free controls (n=17). Participants judged the order of two light flashes presented at different temporal offsets on each of their hands or feet. Slower processing of the flash on one side relative to the other reflects reduced attention to that side of space.

**Results:**

Controls prioritized stimuli on the non-dominant (left) relative to dominant side, consistent with the well-documented normal leftward bias of attention (ie “pseudoneglect”). Regardless of the location (upper or lower limb) of the pain or visual stimuli, people with CRPS showed no such asymmetry, representing reduced attention to the affected side (compared to the greater attention of controls to their non-dominant side). More severe CRPS symptoms were associated with a greater tendency to deprioritize stimuli on the affected side.

**Conclusion:**

Our findings suggest that relative visuospatial bias in CRPS is generalized to the personal space of the affected side of the body, rather than being specific to the personal space of the CRPS-affected limb.

## Introduction

Complex Regional Pain Syndrome (CRPS) is a condition characterized by limb pain, as well as sensory, autonomic, motor, and trophic signs and symptoms.^1^ Studies of people with CRPS have reported distorted perception and mental representation of the affected limb,^2–12^ as well as “neglect-like” symptoms: reduced attention to the affected limb and its surrounding space, relative to the unaffected side, reminiscent of neglect of the contralesional side of space that can follow brain damage.^10^,^13^ This contrasts to the slight leftward bias in spatial attention typical of the general healthy population (referred to as “pseudoneglect”).^14^ The magnitude of neglect-like symptoms in CRPS correlated with greater severity of pain and other clinical symptoms in some studies,^15–22^ but not others.^18^,^23–28^ Therefore, there is a need to better understand how spatial attention is altered in CRPS compared to healthy controls.

Several studies have examined attention bias in CRPS using Temporal Order Judgement (TOJ) tasks. In TOJ tasks, participants indicate the relative order of two stimuli presented one on each side of space with different temporal offsets. Using this method, it is possible to determine if stimuli on one side of space are subject to less attention, and therefore processed more slowly and reported to be ordered second, relative to stimuli on the other side of space (altering their perceived order).^29^ Studies using this method revealed that people with CRPS were slower to process stimuli presented on, or near their affected limb, or in the corresponding side of space relative to stimuli presented on their unaffected side of the body or space.^15^,^16^,^20^,^24^,^26^ That is, they showed a spatial attention bias away from the CRPS-affected side. More recent studies using similar TOJ paradigms, however, found no evidence of any systematic attention bias in CRPS.^18^,^23^,^25^ A possible reason for these mixed findings is that spatial attention biases in CRPS might only manifest, or might be more prominent, under circumstances in which the task-related information is relevant to the participant’s body or specifically the affected limb. Reid et al proposed a “somatospatial inattention” hypothesis, stating that the spatial biases in CRPS are driven by the interactions between spatial and body representations.^16^ This idea was partly inspired by their study using line bisection judgements. People with CRPS showed a bias away from their affected side on a line bisection task only when the line was overlaid on top of their own affected limb or on top of their unaffected limb when it was placed in the side of space where the affected limb usually resides.^16^ Participants showed no bias when bisecting lines that were not overlaid on their limbs.

TOJ studies mostly support the somatospatial inattention hypothesis. Reduced attention to the CRPS-affected limb has been demonstrated using tactile^15^,^16^,^20^ or visual^26^ stimuli delivered directly on the hands (ie in personal space), and using visual stimuli presented in the immediate proximity of the hands (ie in peripersonal space).^24^ However, except for one study,^26^ participants with CRPS showed no spatial bias for visual stimuli presented in the same physical location but when the hands were out of the participant’s view,^23^,^24^ separated from the stimuli by a physical barrier,^25^ for visual stimuli presented at a greater distance from the hands and body, ie in extrapersonal space,^18^,^24^ or for auditory stimuli presented separately to each ear.^16^ That is, in most cases, biased TOJs only occur when judgements are about stimuli presented on or very near to the body, suggesting that visuospatial biases in CRPS are limited to personal and peripersonal space.

If the interactions between spatial and body representations drive spatial biases in CRPS, then spatial biases should be limited to, or stronger in, the affected (versus the mirror symmetric unaffected) body part compared to the other limbs. This is because body representation distortions are typically limited to the affected limb and may be associated with a greater attention bias away from the affected side in CRPS.^18^,^26^ To this end, we used visual TOJ tasks to measure spatial attention in the region of the hands and feet in people with upper or lower limb CRPS, and in pain-free controls. We hypothesized that in people with CRPS primarily affecting one upper limb (“UL-CRPS”) or one lower limb (“LL-CRPS”), attention bias away from the affected side would be limited to or stronger when assessed on the affected versus mirror-symmetric unaffected part of the body compared to the other limbs. That is, UL-CRPS individuals would show a larger attention bias when tested on their hands versus feet, whereas LL-CRPS individuals would show a larger attention bias when tested on their feet versus hands.

## Material and Methods

The study was conducted in accordance with the principles stated in the Declaration of Helsinki, it was approved by the National Health Service Oxfordshire Research Ethics Committee A (ref. 12/sc/0557), and written informed consent was obtained from all participants.

Prior to any data collection, our hypotheses, procedures, and analysis plan were pre-registered at the Open Science Framework (https://doi.org/10.17605/OSF.IO/GY4KC), where the anonymized data and analysis script are also publicly available (https://osf.io/mrf2h).

### Participants

All eligible participants had to have sufficient English language ability to provide informed consent and understand the instructions and could not have any history of neurological disorders (eg stroke, neurodegenerative disease, or traumatic brain injury) or be legally blind. Participants with CRPS had to have received a diagnosis of CRPS type I or II (without or with discrete nerve damage, respectively) primarily affecting one upper or one lower limb at least three months before, and met the Budapest clinical or research diagnostic criteria^1^ at the time of testing. A group of age-matched (±5 years) pain-free controls was recruited to determine whether any biases in the CRPS groups significantly differed from normal performance. These controls could not have experienced pain on most days for three months or more and had to be pain-free at the time of testing. To be able to compare the performance relative to the CRPS-affected versus unaffected side between the CRPS and control groups, we matched the non-dominant side of controls to the affected side of people with CRPS, following the approach used in previous studies.^26^,^30^ A G*power^31^ sample size calculation to detect a medium effect size (*eta^2^* = 0.05) for an interaction between Group and Body Region (see “Statistical analyses” section), with an alpha level of 0.05 and 80% power resulted in a required sample size of 51, with 17 participants per group (UL-CRPS, LL-CRPS, controls). However, we fell slightly short of this recruitment target for the CRPS groups (see “Sample Characteristics” in the “Results” section). The power to detect a medium effect size with the current sample size is 76%.

### Procedures

#### Clinical Assessments

A validated protocol was used to verify that participants with CRPS met the diagnostic criteria and to quantify their overall symptom severity expressed as the CRPS Severity Score (CSS).^32^ As part of this assessment, we used previously reported methods to objectively quantify temperature asymmetry, oedema, and motor weakness.^33^ CSS was not assessed in pain-free controls. All groups underwent additional measures of binocular peripheral vision (ie visual identification task using Landolt C optotypes presented in the periphery)^34^ and mechanical detection thresholds (ie touch detection task using Von Frey filaments)^35^ on hands and feet to explore any visual and somatosensory asymmetries (see Supplementary text and Figures S1 and S2 for detailed procedures and results of these measures).

#### Questionnaires

All participants completed the Edinburgh Handedness Inventory (EHI)^36^ to quantify their relative hand preference at the time of testing (scores < −40 would indicate left-handedness, and >40 would indicate right-handedness). Subjective cognitive representation of the affected/non-dominant limb was assessed using the Bath CRPS Body Perception Disturbance Scale (BPDS),^37^ also in the control group, as this measure was validated only after conception of this study.^38^ Participants with CRPS additionally completed a short-form of the Brief Pain Inventory (BPI)^39^ as a measure of pain intensity and interference. The Tampa Scale for Kinesiophobia (TSK)^40^ was used to measure pain-related fear of movement and re-injury (ie kinesiophobia). Higher scores on the abovementioned questionnaires indicate more severe distortion of body representation (BPDS), pain intensity and interference (BPI), and kinesiophobia (TSK). Pain-free controls did not complete the BPI and TSK measures.

#### Temporal Order Judgement (TOJ) Tasks

The primary outcome was visuospatial attention bias as measured by visual TOJ tasks. In the TOJ tasks, participants were presented with pairs of identical, brief light flashes (10 ms) projected by laser pointers on the dorsal surfaces of their hands and feet (in separate blocks): one light on the hand/foot of the affected (non-dominant for controls) side of the body, and one light on the hand/foot of the unaffected (dominant for controls) side of the body. Pairs of lights were presented with ten different Stimulus Onset Asynchronies (SOAs): ±10, 30, 60, 120, and 240 ms. Negative SOAs represented trials in which the light was presented on the affected/non-dominant side first. Each SOA was repeated 15 times within one block, in pseudo-random order, giving 150 trials per block.

The TOJ tasks were performed in two Body Region conditions. In the “hands” condition, the participants sat with their head in a chin rest viewing a fixation point at 28 cm distance that was aligned with their body midline. Participants’ hands were positioned palms down on the table, either side of the fixation point, with a distance of 9 cm between the fixation point and the center of each hand dorsum. In the “feet” condition, the participants sat with their feet soles down on the floor and leaned forward, with the center of each foot dorsum positioned at a distance of 9 cm either side of a fixation point as in the “hands” condition. The position of their head and gaze fixation were visually monitored by the researcher in the feet condition due to the difficulties of using a chin rest in this body positioning. In separate blocks, each participant reported the perceived order of the two lights under two response conditions: “which light appeared first” and “which light appeared second”, left or right, to control for potential response bias.^41^ Therefore, each participant completed four blocks of TOJ tasks (two Body Region conditions by two response conditions): “hands-first” “hands-second” “feet-first” “feet-second.” Each block was preceded by a short training using only the longest SOAs (ie ±240 ms) with accuracy feedback for every trial (which was not provided in the actual TOJ blocks), and the order of the blocks was counterbalanced with the only restriction that the two blocks of the same Body Region condition were performed consecutively.

### Analyses

#### Data Processing

We re-expressed the data from the “which light appeared second” response condition in terms of “which light appeared first” condition. We also re-expressed the relative number of left/right responses to different SOAs in the TOJ tasks as the number of affected/unaffected responses (CRPS) or non-dominant/dominant responses (controls). We fitted the data with a cumulative Gaussian with the criterion of maximum likelihood to derive an index of spatial attention bias, ie Point of Subjective Simultaneity (PSS). PSS indicates the amount of time by which the light on the affected/non-dominant side should precede or follow the one on the unaffected/dominant side for the two lights to be perceived as simultaneous. Negative PSS indicated a bias away from (ie less attention to) the affected/non-dominant side. We also calculated PSS relative to the left/right side of the body for an exploratory follow-up analysis. In this case, negative values indicated a bias away from the left side. Individual PSS values were averaged across the two response conditions within each Body Region condition (hands and feet).

Although our primary aim and hypothesis concerned spatial bias, indicated by PSS, we also derived Just Noticeable Differences from the TOJ responses to address a secondary hypothesis regarding differences in temporal acuity. Details on the methods, results, and discussion of this secondary analysis are available in the Supplementary text, Table S1, Figures S3 and S4.

### Statistical analyses

To address the primary hypothesis, we conducted a two-way repeated measures ANOVA on the PSS expressed relative to the affected side, with a between-subjects factor Group (UL-CRPS, LL-CRPS, controls), and a within-subject factor Body Region (hands, feet). *A-priori* interaction contrasts, with Holm-Bonferroni correction (*p_adj_*), included comparing hands versus feet in UL-CRPS, hands versus feet in LL-CRPS, UL-CRPS versus controls in the hands condition, LL-CRPS versus controls in the feet condition, UL-CRPS versus controls in the feet condition, and LL-CRPS versus controls in the hands condition. Welch’s approximation of degrees of freedom was used for between-group comparisons. The effect sizes were interpreted according to the suggestions by Cohen (small: *d* ≥ 0.2, *eta^2^* ≥ 0.01, *r* ≥ 0.1; moderate: *d* ≥ 0.5, *eta^2^* ≥ 0.06, *r* ≥ 0.3; large: *d* ≥ 0.8, *eta^2^* ≥ 0.14, *r* ≥ 0.5).^42^ As a follow-up exploratory analysis, we ran the same ANOVA on the PSS expressed relative to the left/right side. Overall, Levene’s tests for the ANOVAs indicated homogenous variances (all *ps* ≥ 0.079).

We also compared the two CRPS groups on CRPS severity (CSS), pain intensity and interference (BPI), body representation distortions (BPDS), kinesiophobia (TSK), and CRPS duration, using independent-samples *t*-tests. In case of significant group differences on any of these factors, we re-analyzed any contrasts (following significant ANOVA effects on PSS) between the UL-CRPS and LL-CRPS groups as ANCOVAs with a between-subject factor Group (UL-CRPS, LL-CRPS) and the relevant variables added as covariates to examine whether they could relate to any differences in attention biases.

Finally, we computed Pearson’s correlation coefficients to explore whether individual differences in the spatial attention bias (PSS) in participants with CRPS were related to any of the abovementioned clinical or psychological characteristics.

## Results

### Sample Characteristics

Sample characteristics are summarized in Table 1. Out of the planned 17 participants per group, 17 controls and 14 CRPS participants per group completed the study before recruitment had to be terminated due to time and resource restrictions. The three groups did not differ significantly in terms of age, sex, and handedness. As expected, either CRPS group reported more distortions in body representation (BPDS) compared to the control group (all *ps* < 0.001), however, there were no differences between LL- and UL-CRPS groups for this measure. UL-CRPS participants had lower EHI scores than control (*p* = 0.013) and LL-CRPS (*p* = 0.001) participants, indicating lower right-hand preference. This difference likely reflects the fact that half of the UL-CRPS participants were affected in their right upper limb, which would reduce their preference for and ability to use their right hand in daily tasks. Compared to UL-CRPS group, the LL-CRPS group contained a higher proportion of people with CRPS type II, showed more severe CRPS symptoms (CSS), reported more intense and interfering pain (BPI) and reported more kinesiophobia (TSK), but there was no difference in CRPS duration.

**Table 1:** Participant Characteristics

	Control (n = 17)	LL-CRPS (n = 14)	UL-CRPS (n = 14)	p*
Age (years)	41.61 [35.39 to 50.67]	44.59 [38.79 to 50.79]	51.55 [41.31 to 58]	0.183
Edinburgh Handedness Inventory [EHI] (/±100)	79.79 [66.14 to 87.71]	88.15 [76.05 to 95.86]	56.72 [34.52 to 75.95]	**0.020**
Handedness (% right)	100%	86%	93%	0.283
Sex (% female)	71%	64%	93%	0.177
Affected side (% left)	NA	71%	50%	0.439
CRPS type (% II)	NA	36%	0%	**0.044**
CRPS duration (months)	NA	30.16 [21.2 to 44.41]	41.94 [28.75 to 70.3]	0.168
CRPS Symptom Severity [CSS] (/16)	NA	12.86 [11.13 to 13.71]	11.13 [9.83 to 12.21]	**0.008**
Pain intensity [BPI] (/10)	NA	6.71 [5.88 to 7.37]	5.14 [4.05 to 5.97]	**0.001**
Pain interference [BPI] (/10)	NA	7.35 [6.54 to 7.88]	4.61 [3.45 to 5.65]	**< 0.001**
Body Perception Disturbance [BPDS] (/57)	12.14 [8.72 to 14.82]	30.93 [23.73 to 36.79]	23.75 [17.79 to 31.47]	0.053
Kinesiophobia [TSK] (/68)	NA	40.79 [35.85 to 44.29]	35.75 [31.71 to 40.24]	**0.025**

**Notes**: Where applicable, maximum possible score on each measure is indicated in the first column in brackets (eg, /10). For continuous factors, mean [bootstrap corrected and accelerated 95% confidence intervals] estimates are reported. *p values from an ANOVA for age and EHI, chi-squared tests for categorical factors, and independent-samples *t*-tests for the remaining continuous factors. *P*-values depicted in bold indicate statistically significant differences between groups. One LL-CRPS participant had missing data for pain intensity and pain interference.

**Abbreviations**: BDPS, Bath CRPS Body Perception Disturbance Scale; BPI, Brief Pain Inventory; CRPS, Complex Regional Pain Syndrome; CSS, CRPS symptom severity; EHI, Edinburgh Handedness Inventory; LL-CRPS, lower limb CRPS; n, number of participants per group; NA, not applicable and not measured in pain-free controls; TSK, Tampa Scale for Kinesiophobia; UL-CRPS, upper limb CRPS.

### Visuospatial Attention (PSS)

**Figure 1:**
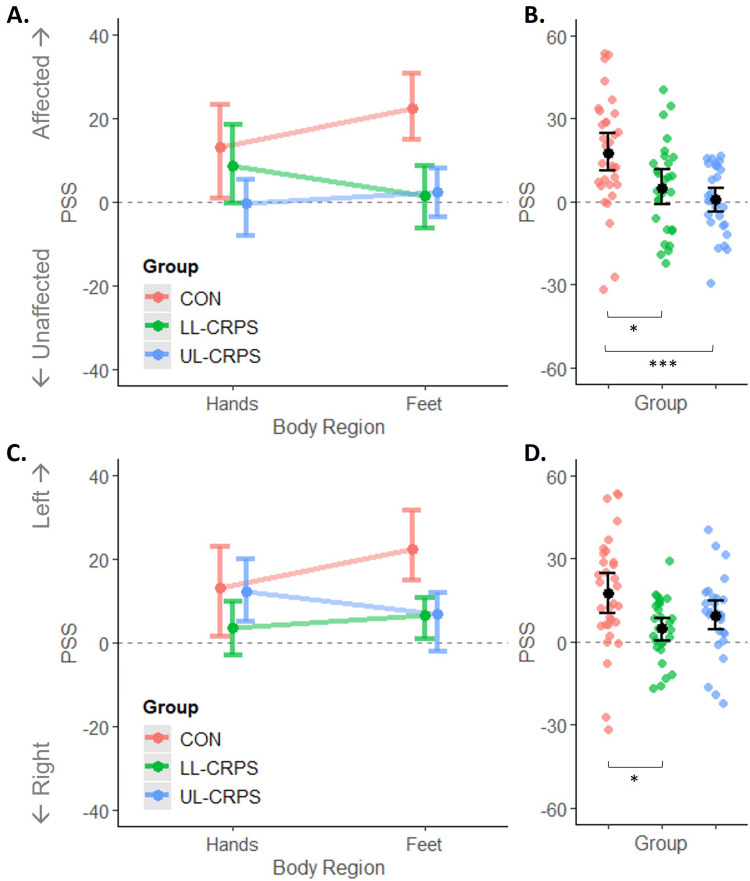
Point of Subjective Simultaneity (PSS) expressed relative to the affected(/non-dominant) versus unaffected(/dominant) side (**A** and **B**), and relative to the left versus right side of the body (**C** and **D**). (**A**, **C**) Interactions between Group (CON, control; LL-CRPS, lower limb Complex Regional Pain Syndrome; UL-CRPS, upper limb CRPS) and Body Region (not statistically significant). (**B**, **D**) Main effects of Group. Smaller dots indicate individual observations (**B**, **D**). Larger dots with error bars represent means with bootstrap corrected and accelerated 95% confidence intervals. One outlier (control) was removed from this figure and the analysis. ***p_adj_ < 0.001, *p_adj_ < 0.05.

The PSS values, indicating the degree of spatial attention bias, are plotted in Figure 1. Results of the ANOVAs are reported in Table 2. One observation within the control group was identified as an outlier on the PSS outcome (value exceeding group mean ±2.5 standard deviations [SD]) and removed from this analysis. For the primary analysis of PSS expressed relative to the affected (/non-dominant) and unaffected (/dominant) side of the body, there was no significant interaction between Group and Body Region (Figure 1A), and no main effect of Body Region. We found a significant large main effect of Group (Figure 1B), where controls had a more positive PSS (stronger bias *towards* the affected/non-dominant side) than LL-CRPS (*t*[58] = 2.66, *p_adj_* = 0.020, *d* = 0.68, 95% CI [0.16, 1.20], moderate effect), and UL-CRPS (*t*[52] = 3.97, *p_adj_* = 0.001, *d* = 1.01, 95% CI [0.47, 1.54], large effect). The two CRPS groups did not differ in their PSS (*t*[50] = 1.09, *p_adj_* = 0.280, *d* = 0.29, 95% CI [−0.24, 0.82]), even when CRPS severity (CSS), pain intensity and interference (BPI), and kinesiophobia (TSK) were included in this comparison as covariates. Retaining the outlier in the analysis produced the same results (Supplementary text, Figure S5, and Table S2).

**Table 2:** Results of the ANOVAs on the PSS Outcome

Effect	*F*	df	*p*	*eta^2^*
**PSS affected(/non-dominant) versus unaffected(/dominant; primary analysis)**				
Group	5.97	2, 41	**0.005**	0.17
Body Region	0.51	1, 41	0.480	< 0.01
Group x Body Region	2.89	2, 41	0.067	0.04
**PSS left versus right (exploratory follow-up analysis)**				
Group	3.82	2, 41	**0.030**	0.11
Body Region	0.98	1, 41	0.329	0.01
Group x Body Region	2.24	2, 41	0.120	0.04

**Notes**: *P*-values depicted in bold indicate statistically significant effects. Row headings in bold indicate the type of analysis.

**Abbreviations**: eta^2,^ effect size; df, degrees of freedom; F, F-test statistic; *p*. p value; PSS, Point of Subjective Simultaneity.

**Figure 2:**
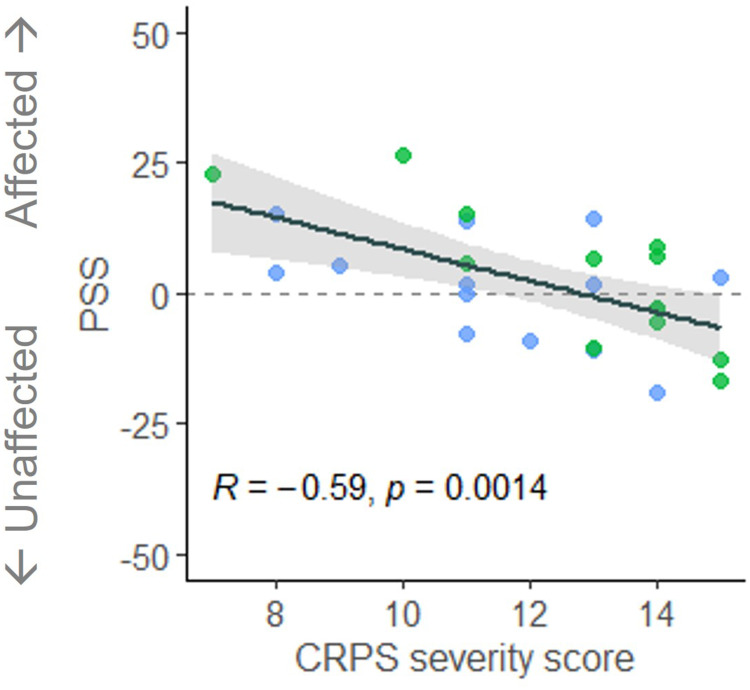
Scatterplot of the relationship between the Point of Subjective Simultaneity (PSS) and the Complex Regional Pain Syndrome (CRPS) severity score, for participants with lower (green) and upper limb (blue) CRPS. PSS is expressed relative to the affected side of the body. Dots correspond to individual observations (PSS averaged across Body Region conditions; two influential observations were removed from this figure and the analysis), with a fitted regression line (black) and 95% confidence intervals (grey shaded area). *R* represents the Pearson’s correlation coefficient with the corresponding *p* value.

To explore relationships between PSS and clinical and psychological characteristics of participants with CRPS, we pooled the data of the LL-CRPS and UL-CRPS groups, and averaged the PSS estimates across the Body Regions, as there were no differences between these groups, nor an interaction between Group and Body Region. CRPS severity (CSS) showed a strong negative relationship with attention bias (PSS; Figure 2), suggesting that more severe symptoms were associated with a larger attention bias away from the affected side (ie less attention to the affected relative to the unaffected side). Data of two participants (one LL- and one UL-CRPS) were identified as influential (Cook’s distance > 0.15)^43^ and had been removed from this analysis, however, retaining them in the data resulted in a consistent but marginally significant correlation (*p* = 0.055; Supplementary Figure S6). PSS was unrelated to CRPS duration, pain intensity and interference (BPI), body representation distortions (BPDS), or kinesiophobia (TSK; *rs* ≤ 0.17, *ps* ≥ 0.39).

As all control participants were right-handed, their results in our primary analysis, ie a stronger bias *towards* the non-dominant side, could be explained by a stronger bias towards the left side (ie pseudoneglect). Therefore, we conducted a follow-up exploratory ANOVA on the PSS expressed relative to the left versus right side of the body, for all participants, in case the results could better be interpreted as attenuation of pseudoneglect in CRPS rather than bias away from the affected side per se.^27^,^44^ Similar to the primary analysis, there was only a significant moderate main effect of Group (Figure 1C and D), which was driven by a more positive PSS (stronger leftward bias) in controls compared to LL-CRPS (*t*[50] = 3.09, *p_adj_* = 0.010, *d* = 0.78, 95% CI [0.26 to 1.30], moderate effect). The difference between controls and UL-CRPS did not reach statistical significance (*t*[56] = 1.83, *p_adj_* = 0.145, *d* = 0.47, 95% CI [−0.04 to 0.98]). There was no significant difference between the CRPS groups (*t*[51] = −1.33, *p_adj_* = 0.189, *d* = −0.36, 95% CI [−0.88, 0.17]) even after including the covariates. Correlation analyses between PSS expressed relative to the left versus right side and clinical and psychological characteristics (CRPS severity [CSS], duration, pain intensity and interference [BPI], body representation distortions [BPDS], or kinesiophobia [TSK]) revealed no significant relationships (absolute *rs* ≤ 0.24, *ps* ≥ 0.22).

## Discussion

In contrast to our hypothesis, we found no evidence that the neglect-like bias in CRPS was greater in the region of hands versus feet in UL-CRPS and in the region of feet versus hands in LL-CRPS. Instead, irrespective of the body region, both CRPS groups showed an absence of the bias exhibited by controls towards their non-dominant (left) side, equating to less attention to the affected relative to the unaffected side of the body. This suggests that attention to personal space is biased in CRPS, regardless of which body part (upper or lower limb) is affected.

Our finding that attention is biased relative to controls regardless of which limb is affected by CRPS could indicate that somatospatial inattention^16^ in CRPS does not result from the overlap of spatial cognition with body representation (which is only distorted for the affected limb) but is instead a spatial bias that specifically manifests within the confines of personal space (which constitutes the entire body). This conclusion could also explain why we found evidence of a significant bias (relative to controls) in people with CRPS in the current study but not in a recent study in which we aimed to directly test the somatospatial inattention hypothesis.^16^ In our previous study, we manipulated the involvement of body representation in a series of spatial attention tasks by using near versus far distance from the body, body-related versus neutral stimuli, and mental rotation of body parts versus neutral objects. However, except for some isolated findings in participants with LL-CRPS, there was mostly no evidence for a body-related spatial attention bias in CRPS.^18^ Unlike the current study, task-relevant information in our previous study was never presented directly in participants’ own personal space (ie directly on the body). When combined with the current study, this suggests that attention bias in CRPS manifests when the task directly involves personal space, but not when only body representation is implied. Our exploratory analysis provides further support for this conclusion by showing that the visuospatial bias was unrelated to self-reported body perception disturbance for the CRPS-affected limb. Despite some previous studies suggesting such relationship,^18^,^26^ it is indeed not systematically found.^18^,^23–28^ Precedence for a specific impairment to attention to personal space is provided by brain-lesioned patients showing personal neglect in the absence of attention biases to other regions of space.^45–47^

An alternative interpretation of the differences that we observed between patients with CRPS and pain-free controls is that people with CRPS showed attenuation of pseudoneglect (ie the leftward bias of attention that is normally seen in non-clinical populations).^14^ Our control participants were all right-handed, therefore, increased attention to the non-dominant (left) side in this group is equivalent to pseudoneglect. When the TOJ responses of participants with CRPS were expressed relative to left versus right, instead of affected versus unaffected side, they showed reduced leftward bias relative to controls. Note that this difference only reached statistical significance in participants with LL-CRPS, and it is unclear why such reduction would be shown only in that group. The few studies that have evaluated attention to left versus right sides of space in CRPS report either no deviations from controls on a range of visuospatial tasks,^23^ or an exaggerated pseudoneglect upon performing robot-assisted line bisections^44^ and in judging where visual stimuli crossed one’s perceived body midline in extrapersonal space.^27^ In healthy, non-clinical populations, pseudoneglect is thought to arise from the right hemisphere dominance for spatial attention.^14^,^48^ Research in older (>60) adults and psychiatric clinical populations^49–51^ suggests that attenuated pseudoneglect might reflect reduced functional cerebral asymmetry in spatial attention, indicating right parietal cortex dysfunction. Further evidence consistent with parietal dysfunction in CRPS, comprehensively reviewed elsewhere,^10^ demonstrated impaired constructional and gnostic abilities, including finger identification and discrimination.^52–55^ Such neuropsychological symptoms are typically found in patients with parietal lesions. Together with altered spatial attention in personal space demonstrated in the current study, and well-documented distortions of body representation in CRPS,^2–9^ both of which have been associated with parietal lobe function,^56–62^ this evidence supports the idea that a disruption of parietal cortical networks could be underlying the cognitive changes in CRPS.^10^,^63^ Nonetheless, in light of the sparseness and inconsistency of previous findings, and our results showing a significant difference from controls only in LL-CRPS when the data are expressed in terms of left versus right bias, we interpret the apparent attenuation of pseudoneglect with caution.

Numerically, the PSS values of participants with CRPS were closer to zero (which would correspond to a balanced distribution of spatial attention) rather than shifted towards negative values (bias *away from* the affected/non-dominant side). However, we interpret them in relation to the PSS of pain-free controls, which was shifted towards positive values (bias *towards* the affected/non-dominant side). This is because normally, in non-clinical populations, the distribution of spatial attention is not balanced, but shifted leftwards (ie pseudoneglect). Therefore, less positive PSS values in participants with CRPS relative to controls are consistent with reduced attention to the affected side of the body. This conclusion is in line with previous TOJ studies in CRPS that tested spatial attention in personal or peripersonal space,^15^,^16^,^20^,^24^,^26^ whereas most studies testing spatial attention in the near space within participant’s reach (but not in the direct vicinity of their body parts), or in the extrapersonal space beyond their reach, reported no evidence for any lateralized biases.^18^,^23–25^ The existing evidence appears to converge on the conclusion that spatial attention of people with CRPS is only biased away from the information on or in the direct vicinity of their affected side of the body. Our exploratory analysis additionally suggests that the magnitude of this bias may be related to the severity of the condition, as attention to the affected side decreased with increasing severity of CRPS symptoms. No such relationship was found with the attention bias expressed relative to left versus right side.

Several limitations moderate our certainty in the discussed findings. First, the CRPS groups were slightly short of our target sample size. While 14 participants per group would still allow to detect a moderate interaction effect regarding our primary hypothesis, the current study may have been underpowered to detect differences in the visuospatial bias depending on the location of CRPS and the visual stimuli if the true effect was smaller. However, a sample of 28 people with CRPS is still comparable to, or greater than in, most previous studies investigating spatial cognition in CRPS that used similar methods.^15^,^16^,^20^,^24–26^ The second limitation is that the hands and feet conditions were not perfectly matched in terms of viewing distance, due to practical constraints of presenting visual stimuli on feet dorsa and maintaining their unobstructed view. Analysis of temporal acuity showed that participants overall had greater precision in their TOJ judgements for the feet compared to the hands condition (Supplementary text, Table S1, and Figure S3), indicating that this condition was likely easier. However, this should not have affected the *spatial bias* (ie PSS). Note that this was also the first study to measure spatial attention in the region of feet in people with CRPS, distinct from previous measures restricted to the regions closer to the upper limbs (such as the surface of the hands, a table, or a computer screen), even when LL-CRPS was involved.^18^,^26^ Third, the groups in the current study were not perfectly matched on handedness, which can modulate lateralization of spatial attention.^64^,^65^ However, lower right-hand preference on EHI in UL-CRPS participants would be expected if the previously dominant arm is affected by CRPS.

## Conclusion

In the existing literature, people with CRPS appear to present with reduced attention to their affected side only under certain conditions, ie when the relevant information occurs in their personal or peripersonal space. Our current findings support this conclusion by demonstrating that relative to pain-free individuals, people with upper and lower limb CRPS pay less attention to visual information on the affected than unaffected side of their body (ie personal space). This visuospatial bias appears to generalize to the entire hemibody ipsilateral to the CRPS-affected limb, although this remains to be tested for body regions other than the extremities (eg face).
